# Chromatin structure in cancer

**DOI:** 10.1186/s12860-022-00433-6

**Published:** 2022-07-28

**Authors:** Meng Wang, Benjamin D. Sunkel, William C. Ray, Benjamin Z. Stanton

**Affiliations:** 1 Center for Childhood Cancer and Blood Diseases, Abigail Wexner Research Institute at Nationwide Children’s Hospital, Columbus, OH 43205 USA; 2 Battelle Center for Mathematical Medicine, Abigail Wexner Research Institute at Nationwide Children’s Hospital, Columbus, OH 43205 USA; 3 Department of Pediatrics, The Ohio State University, Columbus, OH 43205 USA; 4 Department of Biological Chemistry and Pharmacology, The Ohio State University College of Medicine, Columbus, OH 43210 USA

**Keywords:** Chromatin structure, Genome sequencing, Cancer epigenetics, Sarcoma, Structural variation, Chromatin imaging

## Abstract

In the past decade, we have seen the emergence of sequence-based methods to understand chromosome organization. With the confluence of *in situ* approaches to capture information on looping, topological domains, and larger chromatin compartments, understanding chromatin-driven disease is becoming feasible. Excitingly, recent advances in single molecule imaging with capacity to reconstruct “bulk-cell” features of chromosome conformation have revealed cell-to-cell chromatin structural variation. The fundamental question motivating our analysis of the literature is, *can altered chromatin structure drive tumorigenesis?* As our community learns more about rare disease, including low mutational frequency cancers, understanding “chromatin-driven” pathology will illuminate the regulatory structures of the genome. We describe recent insights into altered genome architecture in human cancer, highlighting multiple pathways toward disruptions of chromatin structure, including structural variation, noncoding mutations, metabolism, and *de novo* mutations to architectural regulators themselves. Our analysis of the literature reveals that deregulation of genome structure is characteristic in distinct classes of chromatin-driven tumors. As we begin to integrate the findings from single cell imaging studies and chromatin structural sequencing, we will be able to understand the diversity of cells within a common diagnosis, and begin to define *structure–function relationships* of the misfolded genome.

## Background

The sequencing of the human genome [1] has motivated fundamental questions to understand non-coding components of its heritability. The vast majority of human DNA sequences are located outside the exon regions of the genome, or “exome”. This leads to the question, *is there selective pressure to retain large non-coding regions as physical scaffolding, to provide regulation for genic regions?* Methods to sequence protein-genome interactions *in trans* and long-distance *cis*-chromatin interactions have revealed insights into regulatory functions of non-coding regions through comprehensive mapping [2].

The versatility of high-throughput genome sequencing has enabled mapping of “one-to-all” chromatin interactions with a single “viewpoint” (4C) [3], or with several “viewpoints” (5C) [4]. The sequencing of “all-to-all” chromatin interactions (Hi-C) [5] has been refined with *in situ* approaches to better preserve native chromatin structure [6]. The protein-centric versions of these chromatin sequencing technologies (ChIA-PET, HiChIP, AQuA-HiCHIP) now enable precise quantitative examination of how distinct regulatory factors mediate loops [7–9]. One of the key findings from chromatin sequencing is that *the cancer genome is structurally distinct from human reference genomes*.

We describe evidence for altered chromosome folding in cancer in the context of chromatin interaction domains, and chromosome structural variation in malignancies. Studies of chromatin loops, topologically associating domains (TADs), chromatin compartments, and structural variation (SV) provide evidence for this finding by revealing key elements of altered genome structure in cancer. Thus, we examine genome structure–function relationships in human malignancy, with a focus on alterations in chromatin interaction domains.

## Main text

### Chromatin loops in cancer

In Hi-C data, chromatin loops appear as punctate regions of heightened interactions relative to neighboring chromatin [10, 11]. While *cis*-chromatin sequencing methods including Hi-C enable detection of chromatin loops and long-range interactions, more recent methods, including HiChIP [7, 9], TrAC-looping [12], and Capture-HiC (capture-C) [13], focus on high-resolution sequencing of shorter-range loops at kilobase resolution with increased accuracy. Each of these recent methods attains high proportions of *paired-end tags*, or PETs that are useful for defining chromatin interactions, including functional *enhancer-promoter interactions*.

Substituting the micrococcal nuclease (MNase) enzyme for other cutting enzymes increases resolution in sequencing shorter-range chromatin domains. Excitingly, 3C-based methods and micrococcal nuclease (MNase) have converged in recent methods for high-resolution chromatin structural sequencing, including Micro-C [14] and Micro-capture-C (MCC) [15]. The more recent MCC method resolves proximal enhancer-promoter contacts, within several kb, which has remained challenging for Hi-C at standard sequencing depth. This presents new opportunities to systematically examine altered short-range chromatin interactions in human cancers.

Moreover, the higher-resolution chromatin interactions observed in MCC and Micro-C, also provide context for defining transcription factor (TF) binding sites within chromatin loops. This enables new approaches to understand TF-driven childhood malignancies such as the chimeric oncoproteins that drive rhabdomyosarcoma (RMS) [16] and Ewing sarcoma (EWS) [17]. We anticipate exciting advances in the years ahead in the precise determination of localization of oncogenic TF-chimeras in the context of chromatin looping.

Recent studies in RMS, a rare pediatric soft tissue cancer, have revealed context-specific roles for chromatin looping. In the RMS subtype driven from TF-chimeras, termed fusion-positive (FP-RMS), there is evidence that the PAX3-FOXO1 oncoprotein has pioneer activity [16]. The intrinsic ability of this chimeric TF to alter repressive chromatin states enables a network of chromatin interactions in FP-RMS, including looping at the *MYOD1* and *SOX8* gene loci to promote positive autoregulation of tumor-specific gene activation [18]. The clinical molecule entinostat, which inhibits the function of histone-H3 deacetylases, systematically alters chromatin looping in FP-RMS preceding myogenic differentiation and loss of tumor proliferation [9, 19]. With spike-in quantification of HiChIP (AQuA-HiChIP), we observed that entinostat treatment has immediate-early effects to augment chromatin looping in FP-RMS, deregulating gene expression [9, 18]. In another major subtype of RMS, termed fusion-negative (FN-RMS), chromatin looping stabilizes expression of the pseudo-oncogene *SNAI2* [20]. The essential TF, MYOD, drives RMS in each major subtype, through induced gene expression and through chromatin organization, observed through HiChIP [18, 20]. The clinical RAS inhibitor, trametinib, inhibits ERK activity and suppresses expression of *SNAI2*, promoting FN-RMS tumor differentiation [20, 21]. Determining the chromatin architectural functions of RAS activity in FN-RMS will be of high interest. Taken together, there are distinct parallels between MYOD associated looping events in FP-RMS and FN-RMS, each of which can be altered with clinical or pre-clinical molecules. Studies to determine the precise regulatory influences of pioneer TFs on chromatin looping in sarcomas and other childhood tumors will likely illuminate general principles of chromatin domain dysregulation in aggressive cancers.

**Fig. 1:**
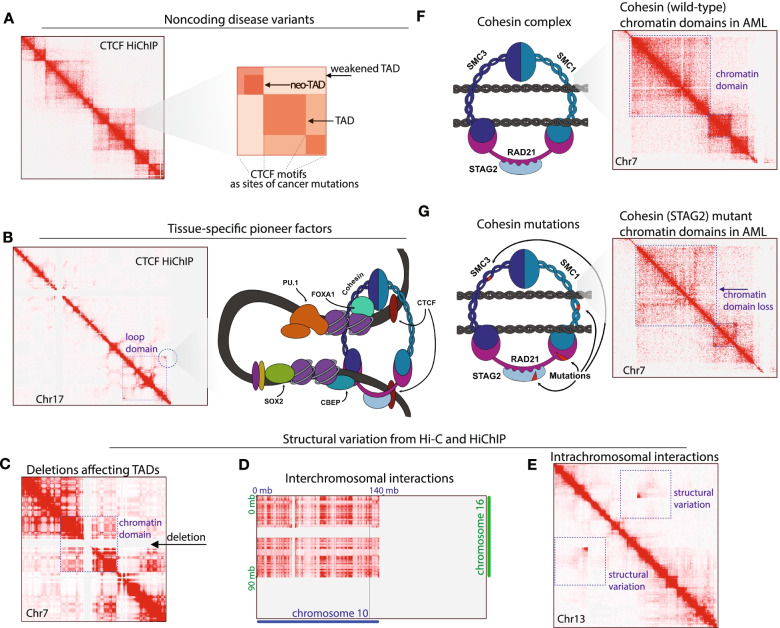
Major chromatin structural attributes of cancer. **A** Disease variants are associated with chromatin interactions. We illustrate non-coding mutations affecting CTCF binding sites in the context of weakened TADs, neo-TADs, and TAD boundaries. CTCF HiChIP data visualized (from [85]) with annotations for how these structural elements may be altered in tumors. **B** Tissue-specific pioneer transcription factors at loop anchors. We illustrate chromatin loop domains, visualized (from [85]) with CTCF HiChIP, and annotations for how transcription factors would occupy the termini of loop domains. **C** Structural variants can alter chromatin domains. We illustrate how deletions alter the visualization of TADs and chromatin domains. Data visualization is from IMR90 cells [6]. **D** Illustration of interchromosomal rearrangements revealed in in Hi-C experiments, with interactions spanning chromosome 10 and chromosome 16 from GM12878 cells, visualized from available Hi-C data [6]. **E** Intrachromosomal structural variation with rearrangements occurring within a chromosome, viewed from HiChIP experiments in AML cells, focused on chromosome 13 [85]. **F** Mammalian cohesin complexes are illustrated with the major subunits SMC1, SMC3, RAD21, STAG2 shown (left). Chromatin domains are shown in AML cells with wild-type cohesion complexes (right; chromosome 7), visualized from available data [85]. **G** Cohesin mutations affect chromatin interactions. Cohesin loss can occur through alterations in the major subunits, as shown (left). The result of Cohesin mutations on chromatin interactions is the substantial loss of TAD-level interactions, as visualized from available data on AML cells with STAG2 loss (right; chromosome 7, [85])

The motifs of TFs influence regulatory chromatin looping in human cancer. Recent studies reveal that highly penetrant noncoding genetic variants have the potential to affect chromatin interactions. Massively parallel TF-motif binding assays coupled to sequencing have revealed the specificities for disease-causing DNA mutations that alter the ability of TFs to recognize their motifs [22]. A capture-C study in human breast cancer demonstrated that TF-motif pairs were altered at regulatory loci encoding disease SNPs [23]. These regulatory SNPs associated with altered chromatin loops in breast cancer were associated with pioneer factors (FOXA1, GATA3) and estrogen receptor (Fig. 1A,B) [24]. Moreover, altered chromatin looping was found to occur at loci encoding major oncogenes (*MYC*) and tumor suppressors (*CDKN2A*). Conceptually, these findings motivate examining altered enhancer interactions in cancers. Analyzing CTCF binding sites in human cancers reveals recurrent mutations and deletions of these motifs in leukemia (T-ALL), esophageal tumors, and liver cancer [25].

Experimental evaluation of many of these CTCF motif alterations with a method called ChIA-PET [25], which enables sequencing protein-centric loops, indicates functional consequences for chromatin interactions. Key loci encoding genes required for tumor proliferation are found in regions associated with altered loop anchor sites. Observations of noncoding variants in human cancers motivated systematic analyses of altered chromatin loops in cancer cell lines, using Hi-C and ChIA-PET [26]. Similar to the Baxter et al. study [23], Snyder and co-workers observed cell-type specific pioneer factors at chromatin loop anchors (e.g., PU.1), and enrichments for penetrant noncoding disease variants at loops [26]. In a recent study on subtype-specific chromatin states in bladder cancer, the pioneer factors FOXA1 and GATA3 were each found to serve as “loop anchors” [27]. Providing further conceptual links between pioneer factor function and 3D genome structure, a recent report demonstrated that *GATA3* gene expression levels can alter chromatin architecture in leukemia, and that polymorphisms in *GATA3*’s intronic regulatory sequences could impact its expression [28]. These studies motivate hypotheses that noncoding cancer mutations might be disrupting chromatin loop structures, and altering binding of tissue-specific TFs or pioneer factors at loop anchors (Fig. 1B).

Recent evidence indicates that chromatin domains can be targeted by clinical [11] or pre-clinical [9, 29] chemotherapeutic strategies. New insights into the regulatory roles of topoisomerases have revealed the potential of the clinical molecule etoposide to covalently disrupt chromatin domains [11]. The conceptual advance highlighted by a chemotherapeutic agent targeting torsional stress associated with *cis* chromatin interactions suggests that altered chromatin domains might serve dual roles as drivers and vulnerabilities in human cancer. This leads to the question, *what are the characteristics of chromatin contact domains in cancer?* We explore this question, in the context of driving alterations in key gene classes, and structural variation in cancer genomes.

### Structural variation and chromatin domains

Recent comparative studies in whole genome sequencing (WGS), chromatin sequencing, and imaging methods, have revealed that Hi-C, especially in combination with whole genome sequencing, can be extremely powerful in identifying structural variation (SV) [30, 31]. From studies of SV and chromatin architecture (reviewed, [32]), it is becoming clear that Hi-C represents an efficient approach for *de novo* detection of SVs in cancer genomes (Fig. 1C,D,E). The impact of these studies will be transforming in several key areas. New insights into how focal deletions, inversions, and translocations are systematically altering the regulatory functions of enhancers or insulators will provide connections between gene regulation and structural variation. Additionally, topological context for copy number variation (CNV) and gene-fusion events in cancer will reveal how alterations reside within chromatin domains. Examining the effects of SVs on the non-coding genome as well as the impact on coding regions will continue to illuminate epigenetic mechanisms driving tumors.

A recent study has revealed that in leukemia genomes, SV modifies the proximity of the *BCL11B* gene locus and its enhancer, thereby driving its expression in progenitor cells [33]. The authors mapped HiChIP data from leukemia samples onto patient-specific reference genomes to account for the SV present. The recurrent translocations impacting the *BCL11B* gene locus were found to frequently involve transposition of enhancer elements that produced functional consequences in gene expression. The study also revealed enhancer-specific CNV (enhancer amplification) affecting *BCL11B* gene regulation. Thus, through structural repositioning, or amplification of enhancers, leukemia gene regulation is systematically altered. Recently, shallow Hi-C approaches have helped define SVs leading to *ETV6-RUNX1* gene fusion events in leukemia, and have revealed new patterns of potential chromothripsis (a series of multiple catastrophic chromosomal rearrangements) [34, 35]. It is of note that lower resolution methods and exome-focused methods like SNP arrays or RNA-seq, may not efficiently capture information regarding chromothripsis, while 3D chromatin sequencing may be more efficient for identifying these patterns of SVs. We anticipate further utility of spike-in normalized chromatin architectural sequencing in the context of chromosomal imbalances (e.g., aneuploidy), which occur in as much as 90% of human tumors [36]. Studies of childhood cancers which rarely exhibit signatures of high mutational frequencies but often display signs of chromothripsis [37, 38] may benefit from these new approaches.

In diffuse intrinsic pontine glioma (DIPG), CNV affecting tumor-specific gene expression of *TCF12* and amplification of its enhancer have been observed in Hi-C studies [39]. In another recent study, enhancers subject to SV were shown to drive expression of *MYC* in lymphoma, through translocation events [40]. In bladder cancers, where *GATA3* and *FOXA1* may have characteristic altered gene expression, Hi-C has been used elegantly to detect patterns of CNV and SV [27]. In hematologic tumors, and solid tumors, SV-induced enhancer transposition can regulate the expression of oncogenic drivers through proximity. The increased usage of low depth Hi-C or HiChIP to elucidate patterns of SV or altered enhancer function will be impactful across the clinical and basic sciences.

The developmental consequences of SVs on altered chromatin domains can be severe, with altered gene expression patterns resulting from de novo TAD formation (“neo-TADs”), TAD-fusion events, and altered boundaries (reviewed [32]) (Fig. 1A,C,E). In comparative studies of cancer 3D genomes, Yue and colleagues uncovered SVs which alter chromatin interactions in prostate, breast, gastric, tumors and hematologic tumors [41]. Recurrent alterations in *cis*-chromatin interactions were observed at loci encoding the pioneer factor FOXA1 (prostate cancer), the cell cycle gene *CDK12* (breast cancer), and the *RAB36* gene (leukemia). Interestingly, RAB36 is frequently observed within a conjoined chromatin contact domain resulting from SV. Yue and colleagues observed that *RAB36* gene expression was associated with poorer patient outcomes, linking SV-mediated chromatin domain alterations with disease etiology. We propose that the developmental alterations in gene expression patterns derived from SV-altered chromatin domains are highly relevant in human cancers, and we anticipate exciting advances to in this area in coming years.

There is evidence that SVs occurring in human cancers are frequently more complex than in other tissues, and this has implications for *cis-*chromatin interactions. A recent comprehensive analysis of SV in human cancer observed recurrent enhancer-deletions for loci encoding tumor suppressive genes, and recurrent *de novo* TAD formation enabling oncogene expression[30]. Interestingly SVs are also a strikingly common feature across the spectrum of human tumors, but many Mb-scale SVs are challenging to define with short-read sequencing alone [42]. However, short-read genome sequencing data could be used to construct subtype-specific reference genomes, which would allow more accurate SV identification using 3D sequencing data. It is of note that the overall frequency of SV occurrence is positively associated with the accessibility of local chromatin states in cancers, suggesting that euchromatin might be predisposed to these alterations.

In distinct cancers, there are common “SV pathways” toward recurrent fusion-oncogene events, while a diversity of SV types may result in amplification of common oncogenes or losses of major tumor suppressors [42]. Increasing evidence supports an association between unique cancer types and idiosyncratic SV patterns, linked to altered chromatin domains [43]. One important aspect of this, is that distinct tumors might have recurrent alterations in chromatin domain boundaries, linking SVs to gene mis-regulation including deletions, interchromosomal rearrangements and intrachromosomal variation (Fig. 1C,D,E). Understanding the major chromatin architectural drivers of human cancers will require integrating SVs in the context of repurposing transcriptional regulatory elements and domains. We anticipate definitions of hallmarks of architectural drivers of cancer as we learn increasingly about the recurrent patterns of domain alterations induced from SVs.

### Cancer metabolism and cis-chromatin interactions

Increasing evidence has revealed chromatin structural phenotypes driven from recurrent cancer mutations in genes encoding metabolic regulators. Two major classes of metabolic mutation that each alter the Krebs cycle are highly penetrant in human tumors. In each case, toxic accumulations of metabolites result in differentially methylated regions (DMRs) across the genome. However, the *SDH* class and *IDH* class of oncogenic mutations rely on distinct mechanisms to induce their convergent effects on the epigenetic state of the cell. One particular class of these penetrant mutations renders the SDH-family enzymes catalytically deficient, which results in accumulation of succinate before it can be processed. High levels of succinate can inhibit several classes of demethylase enzymes, including TET-family and JMJD-family demethylases, thus increasing methyl-CpG content [44], augmenting chromatin succinylation [45], and increased histone H3K9-methylation [46]. Moreover, recent studies suggest that aberrant succinylation levels may also augment the placement of H3K4me3 at loci encoding cell-type specific regulatory genes [47].

Connecting altered CpG methylation and altered tumor metabolism, a recent report revealed DNA hypermethylation at CpG islands in IDH-driven gliomas [48]. Importantly, CTCF binding sites were associated with these DMRs. With evidence that CTCF binding anchors genomic looping, [49] these observations motivated chromatin structural studies. Strikingly, approximately half of the DMRs occurring at CTCF sites overlapped with chromatin loop anchors [44]. Key chromatin contact domains were disrupted, included at the *FGF4* locus, and *KIT* insulator elements. The altered DMRs at these loci resulted in deregulated gene expression for these two GIST drivers. These could be targeted as vulnerabilities with clinical FGFR4, and KIT inhibitors.

A related class of metabolic cancer mutations in the IDH enzyme has also been reported as a driver in leukemias and gliomas [50]. Similar to SDH mutations, IDH mutations induce de novo DMRs through accumulations of metabolites, α-ketoglutarate and most notably 2-hydroxyglutarate, that can inhibit TET-family enzymes and histone demethylases. Interestingly, gene pairs spanning TAD boundary junctions are highly sensitive to IDH mutational status, suggesting that altered CTCF binding may be associated with sensitized DMRs [48]. The IDH glioma insulator-loss mechanism results from methylation-sensitive defects in genomic binding of CTCF, enabling aberrant chromatin domains to drive gene oncogene expression, including *PDGFRA* [48]. Studies of chromatin structure in IDH/SDH-mutant tumors highlight that while altering chromatin domain structures can have subtle or context-specific effects on transcription [10], identifying key alterations in tumor-specific gene expression can lead to targetable vulnerabilities.

While metabolic products can alter chromatin structure–function relationships through enzymatic processes, there is evidence that non-enzymatic processes link metabolic outputs and chromatin structure as well. With new insights from non-enzymatic covalent histone modifications (NECMs) [51, 52], there are additional opportunities to (1) expand the scope of known chromatin PTMs, and (2) interrogate the recently discovered metabolic drivers of NECMs to ask if they have instructive effects on chromatin structure. Examples include evidence for histone glycation [53], histone acylation [54], and histone lipidation [55]. With evidence of altered metabolism [56] and oxidative stress [57] in human cancers, we anticipate exciting advances in the coming years to conceptually relate non-enzymatic histone PTMs with genome structure.

### Imaging and chromatin structure

Bulk-cell genomics has revealed internally consistent principles for contact domains, compartments, and loops. However, single cell imaging sometimes yields distinct or complementary answers to the questions of genome organization. While there is a diffraction-limiting feature in traditional imaging experiments on the order of the visible wavelengths of light (~ 200 nm diffraction limit), 3D-STORM imaging approaches 20 nm resolution [58]. This increased resolution enables characterization of fine chromatin structural features in single cells. Where traditional sequencing-based methods are better equipped for detection of paired *cis*-chromatin interactions, imaging-based methods can capture multi-locus interactions. With 3D-STORM based studies, Zhuang and co-workers examined the cohesion-dependence of domain organization in single cells. With rapid RAD21 depletion [10], the authors observed a statistical retention of chromatin domain structures, suggesting that cohesin plays a primary role in noise-reduction for coherence of contact domain maintenance in bulk cell populations [59]. Similar results, revealing cohesin-independence for contact domains, have been observed with measurements of “globularity” of chromatin domains with super-resolution imaging and cryo-EM [60]. In recent work from Cavalli and colleagues, super resolution microscopy enabled definition of significantly decreased intra-TAD chromatin interactions in the absence of cohesin complexes [61]. Thus, results from bulk cell chromatin sequencing and single cell super resolution microscopy each suggest roles for cohesin function in chromatin interactions within TADs. Also of note was the finding that CTCF loss enables increased inter-TAD chromatin interactivity in single cells [61]. Thus, while stereotypic TAD architecture defined in bulk cell Hi-C and next-generation imaging might differ, key fundamental properties of cohesin and CTCF are conserved at the single cell level [10, 49, 61].

In recent advances, Boettiger and co-workers have reported reconstruction of chromatin interaction domains from high resolution imaging, optical reconstruction of chromatin architecture (ORCA), providing new insights about TAD function and transcription during development [62]. With ORCA, it was found that developmental gene transcription correlates well with chromatin domain formation. ORCA thus helps overcome challenges in characterizing associations between chromatin domain formation and nascent RNA transcription that are problematic for bulk cell sequencing approaches [10]. The resolution of ORCA enables quantification of interaction distances within and across domains. While results from ORCA indicate that active chromatin compartments are correlated with RNA transcription, promoter-enhancer proximity within a single cell is not strongly predictive of transcriptional state. One possible explanation is that within repressed chromatin regions, long-range contacts can still occur [63, 64] while inappropriate enhancer loops might not be productive for initiation [62]. The early results from ORCA in developing drosophila embryos also integrate conceptually with observations from pluripotent cells [65], where domain boundaries are highly sensitive to CTCF positioning and H3K27me3. Understanding locus-specific contexts for CTCF function in single cells, as an insulator for repressive and active chromatin domains will be of high interest. Disruption of heterochromatin is a common feature observed in high resolution imaging studies modeling human cancer progression. ORCA has also revealed dependencies for spatial HOX gene de-repression in recent reports of loss-of-function mutations in mammalian SWI/SNF chromatin remodeling complexes [66]. These studies provide important context for mechanisms of loss of epigenetic tumor suppression with altered SWI/SNF complexes. In recent studies in conditional tumor mouse models, Xu and colleagues observed chromatin restructuring during the course of tumor progression [67]. Consistent with other studies [68], H3K9me3 was observed at DNA repeat elements in the chromatin fiber, while tumor progression resulted in systematic loss of chromatin compaction and altered folding at these regions.

Defining the fundamental connections between deposition of heterochromatin marks and chromatin folding in human cancer will be impactful in coming years. Moreover, applications of ORCA and 3-D STORM imaging to understand conserved properties of chromatin domains in cancers will be highly impactful, as these methods can take into account cellular heterogeneity in human tumors, as well as defining RNA transcription in the precise cellular context in which the chromatin structure is measured. Moreover, integrations with single cell Hi-C (sc-HiC) [69–71] and high-resolution imaging will be impactful. With sc-HiC, it is possible to determine genome structural components in the context of developmental and cell-cycle transitions [71]. It will be exciting to see the synergy between single cell imaging and chromatin sequencing approaches in the coming years in the context of human cancers.

### Cohesinopathies

An important epigenetic pathway towards architectural dysregulation is the mutation of genes encoding major drivers of genome structure, cohesin complexes [10]. The Cancer Genome Atlas (TCGA) sequencing efforts have revealed recurrent cohesin subunit mutations in cancers of the blood system [72]. Of these mutations, penetrance can occur with alterations to the cohesin “motor” subunit (RAD21), for genes encoding structural or scaffolding subunits (e.g., *SMC1A*, *SMC3*), and for the genes encoding associated *STAG1/2* proteins. The class of hematologic “cohesinopathy” connects basic studies of chromatin looping [10, 73], with tumor biology and studies of the differentiation blockade in human malignancy (Fig. 1F,G). The genetics of cohesinopathy provide evidence for *RAD21*, *STAG2*, *SMC1A*, and *SMC3* as driver mutations in acute myeloid leukemia (AML), chronic myeloid leukemia (CML), and pre-leukemic states (myelodysplastic syndrome; MDS) [74].

While cohesin mutations are frequently loss-of-function, it is of high interest to characterize rare sub-classes of cohesinopathy resulting in gain-of-function function rather than haploinsufficiency or total loss (as for X-linked *STAG2*, *SMC1A* mutations). Of note, cohesinopathy mutations have high variant allele frequency, and are considered as founder- or driver-events in leukemic tumor evolution [74–76]. Understanding precise mechanisms for chromatin structural dysregulation as early events in tumor evolution will be impactful. Early reports established that *STAG2* or *SMC3* mutations occur in MDS or de novo AML, supporting the role of cohesin loss in early, driving events in leukemogenesis [77]. With noted roles of requirements of cohesin for chromosomal organization in cell division [78], the functional consequences of early alterations in these complexes in leukemias are not mechanistically linked to significant SV or genome instability [79]. Excitingly, recent studies have also implicated *STAG2* alterations in chromatin structural phenotypes in EWS [80, 81].

It is of high interest that cohesin mutations are often mutually exclusive with *TP53* mutations in AML. This bears similarity to the mutually exclusive relationship between mutations in mammalian SWI/SNF and *PTEN* or *TP53* mutations in human cancers [82]. This mutual exclusivity, or lack of cooccurrence suggests roles for cohesin complexes as major tumor suppressors [83]. To understand the precise roles of cohesin in myeloid malignancy, Levine and co-workers developed conditional alleles for subunits *STAG1* or *STAG2* under the control of the Mx1 promoter [84]. They observed that the loss of STAG2 protein results in the expansion of undifferentiated leukemic progenitor cells in mouse models. The authors asked questions about the dependencies on STAG2 and STAG1 for leukemic transcriptional programs, and found key dysregulated genes, despite an overall low occurrence of statistically altered gene expression. With *STAG2* conditional deletion, there are fewer than 200 statistically altered transcripts, which agrees with previous reports decoupling the role of cohesin function from RNAP2 [10]. However, of the altered transcriptional targets, the overall effect is reminiscent of losses in myeloid differentiation and gains in genes associated with leukemic stemness. Integrating DNA accessibility with these findings, the authors observe losses in pioneer factor PU.1 motifs, alterations in key TAD-boundaries, and altered CTCF motif densities concomitant with STAG2 loss.

Recent studies have also implicated STAG2 loss in lower chromatin contact frequencies within TADs and loops (cf., Fig. 1F,G) [85]. In HiChIP experiments, the authors find that STAG2 loss confers decreased chromatin looping associated with loci encoding leukemia drivers. *De novo* or altered chromatin looping in a STAG2-deficient background induces relative upregulation of key genes within the *HOXA1-HOXA7* region of the *HOX* gene cluster and general losses of expression of *HOXA9*-*HOXA13*. Interestingly there is evidence from several studies regarding compensatory STAG1 activity in STAG2-deficient leukemia, which might result in altered cohesin processivity and a shift from smaller chromatin domains to larger domains. Further studies will be critical to understand the compensatory roles of STAG1/2, and mechanisms of STAG2-mediated maintenance of contact domains for transcription. With recurrent cohesin mutations as drivers of altered chromatin architecture in CML, AML, and EWS, it will be of high interest to understand the commonalities and distinctions in genome structure–function relationships in these tumors.

## Conclusions

We have examined four major areas of architectural dysregulation in the context of human cancer. These common structural tumor drivers are (1) frequent noncoding mutations at chromatin loop anchors and domain insulators, (2) altered TF binding at sites of chromatin interaction, (3) structural variation resulting in domain redistricting, and (4) mutations in cohesin and metabolic genes, upon which chromatin structure is heavily reliant. In each case, further work will be required to establish causality of the chromatin architecture in tumorigenesis. New technology to enable sequencing of altered chromatin domains in human cancer (e.g., long-read sequencing, Hi-C, AQuA-HiChIP) and next-generation imaging of chromatin domains (e.g., 3D-STORM, ORCA) will allow for integration of 3D sequencing and microscopy to define common structural drivers. We anticipate that connections between chromatin structural alterations and patient outcomes will ultimately influence clinical decision making. For example, for low mutational burden tumors with high SV illuminated through 3D genomics, radiation therapy may not be the most efficacious strategy [86]. We look forward to many exciting advances in the coming years with increased integration of single cell imaging approaches and 3D chromatin sequencing to understand chromatin structure in cancer, and to separate cause from consequence in altered chromatin domains.

## Data Availability

Not applicable.

## Abbreviations

3C: Chromosome conformation capture
RMS: Rhabdomyosarcoma
EWS: Ewing sarcoma
FP-RMS: Fusion-positive rhabdomyosarcoma
FN-RMS: Fusion-negative rhabdomyosarcoma
AML: Acute myeloid leukemia
CML: Chronic myeloid leukemia
MNase: Micrococcal nuclease
SV: Structural variation
ORCA: Optical reconstruction of chromatin architecture
cryo-EM: Cryo-electron microscopy
TF: Transcription factor
cohesinopathy: Tumor resulting from mutations in genes encoding cohesin subunits
SNP: Single nucleotide polymorphism
DMR: Differentially methylated region
